# Successful Treatment of Severe Atopic Dermatitis with Calcitriol and Paricalcitol in an 8-Year-Old Girl

**DOI:** 10.1155/2018/9643543

**Published:** 2018-06-24

**Authors:** Christina Bothou, Alexis Alexopoulos, Eleni Dermitzaki, Kleanthis Kleanthous, Anastasios Papadimitriou, George Mastorakos, Dimitrios T. Papadimitriou

**Affiliations:** ^1^Division of Endocrinology, Diabetes and Metabolism, Medical Department 1, University Hospital, Goethe University, Frankfurt am Main, Germany; ^2^Pediatric Dermatology Clinic, 1st Department of Pediatrics, Aghia Sofia Children's Hospital, University of Athens, Athens, Greece; ^3^Department of Pediatric, Adolescent Endocrinology & Diabetes, Athens Medical Center, Athens, Greece; ^4^Division of Pediatric Endocrinology, 3rd Department of Pediatrics, Attikon University Hospital, Haidari, Athens, Greece; ^5^Endocrine Unit, Second Department of Obstetrics and Gynecology, Aretaieion Hospital, Medical School, University of Athens, Athens, Greece

## Abstract

Atopic dermatitis (AD) is a chronic inflammatory disease affecting children and adolescence. The traditional therapeutic options for AD, including emollients topically and immune modulatory agents systemically focusing on reducing skin inflammation and restoring the function of the epidermal barrier, are proven ineffective in many cases. Several studies have linked vitamin D supplementation with either a decreased risk to develop AD or a clinical improvement of the symptoms of AD patients. In this report, we present a girl with severe AD who under adequate supplementation with cholecalciferol was treated with calcitriol and subsequently with paricalcitol. She had significant improvement—almost healing of her skin lesions within 2 months, a result sustained for more than 3 years now. Because of hypercalciuria as a side effect from calcitriol therapy, treatment was continued with paricalcitol, a vitamin D analogue used in secondary hyperparathyroidism in chronic kidney disease. Calcitriol therapy may be considered as a safe and efficacious treatment option for patients with severe AD, particularly for those with refractory AD, under monitoring for possible side effects. Treatment with paricalcitol resolves hypercalciuria, is safe, and should be further investigated as an alternative treatment of atopic dermatitis and possibly other diseases of autoimmune origin.

## 1. Introduction

Atopic dermatitis (AD), also known as atopic eczema, is a common and chronic inflammatory skin disease characterized clinically by pruritus, erythematous lesions, and xerosis with a specific age distribution. AD usually starts in early childhood and affects up to 20% of children and up to 3% of adults with an increasing prevalence over the last years. AD often coexists with other allergic conditions, such as asthma and food allergies. AD may have a great impact on the quality of life for the patients and their relatives, depending on the extent of the clinical manifestations; therefore, it is essential to be effectively treated [1].

Atopic dermatitis is a condition that requires interplay from several factors to explain its pathogenesis. Defects in the epidermal barrier because of filaggrin deficiency or decrease of other protective factors in conjunction with environmental factors result in the formation of acute lesions. These lesions become chronic because of the immune system activation and consequently the dysregulation of various types of immune responses. Additionally, a decreased count of antimicrobial peptides, like beta-defensins and cathelicidins, result in bacterial colonization. Activated dendritic cells by thymic stromal lymphopoietin from keratinocytes and by antigens stimulate proliferation of T-helper 2 (Th2) cells, which secrete inflammatory cytokines that worsen AD severity, such as interleukin 17 (IL-17) [2].

The traditional therapeutic options for AD include the use of emollients topically, to restore the epidermal barrier function. For acute flares, the first-line treatment remains the local use of corticosteroids [3], simultaneously with local calcineurin inhibitors, to maintain remission. In some cases, narrowband ultraviolet B phototherapy, normally not recommended for children <18 yrs, has been proven effective [4], which implies a possible role of UV-B in increased local 1–25 OHD3 Vit D production [5], among other possible mechanisms involved. Oral corticosteroids in conjunction with nonspecific immunosuppressive drugs are used for the more severe cases. These classic treatments are focused on reducing skin inflammation and restoring the function of the epidermal barrier. However, in many cases and especially in children, these therapies are proven inadequate [6].

The function of vitamin D (Vit D) is traditionally related to the regulation of calcium and phosphate homeostasis, which is essential for bone formation and resorption. However, over the last years, the functions of Vit D related to cardiovascular, neoplastic, microbial, and autoimmune diseases are under continual investigation, basically because of the significant anti-inflammatory and immunomodulatory properties of this nuclear receptor-activating hormone. Older as well as recent data have shown that vitamin D affects immune mechanisms, keratinocytes, and subsequently the skin barrier function [7, 8]. More specifically, keratinocytes express vitamin D receptor (VDR) and so they respond to the fully active form of vitamin D, the 1–25 OHD3 Vit D (calcitriol), expressing involucrin, transglutaminase, loricrin, and filaggrin, and producing antimicrobial peptides (AMPs). Vit D is the most potent regulator of the epidermal differentiation, at the level of both gene expression and mRNA stability [9].

There are few studies which have suggested that vitamin D supplementation may be a safe and effective alternative treatment for AD, considering that Vit D deficiency is related to the severity of the AD [10, 11]. Worm et al. recently showed that oral Vit D decreases the T-cell-dependent cytokine production, including IL-17 [12].

Oral paricalcitol, the 19-nor-1,25(OH)2D2 analog of Vit D, is 3 times less potent compared to calcitriol regarding VDR activation but displays a 10-fold decreased effect on intestinal calcium absorption and bone mobilization [13], making it a safer alternative for VDR activation as far as risk for hypercalcemia and/or hypercalciuria is concerned. Paricalcitol is used in the treatment of secondary hyperparathyroidism in chronic kidney disease in children [14], as well as in adults, but there is no current evidence in the literature regarding other possible uses of oral paricalcitol.

After written informed consent, we present the case of a girl with severe atopic dermatitis who had showed no improvement despite the use of all the already existing therapeutic modalities for AD in her recent past.

## 2. Case Presentation

**Figure 1:**
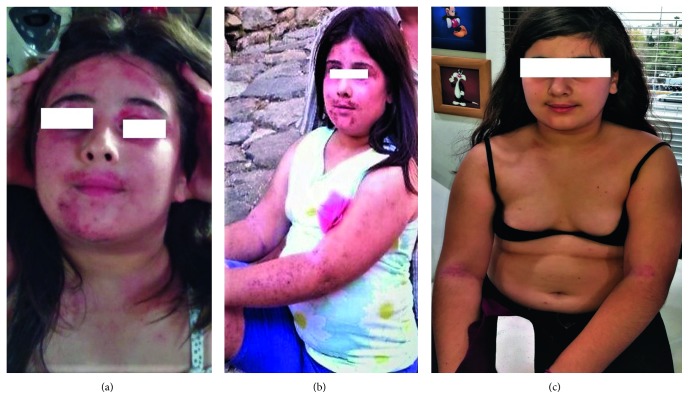
(a) First presentation, before calcitriol therapy; (b) at 2 months already healing; (c) at 3 years after the first presentation, currently in paricalcitol therapy, with minimal lesions.

An 8-year-old girl was referred for investigation of hyperthyrotropinemia (increased TSH) and cushingoid features probably due to oral prednisolone intake prescribed for the management of her AD. The patient was prepubertal and overweight, with a body mass index (BMI) of 26.56 kg/m^2^. Already by the age of 6 months, our patient was diagnosed with mild atopic dermatitis which was successfully managed with emollients. As a toddler, atopic dermatitis relapses became more frequent, but the episodes were still easy to control with topical emollients and corticosteroids. Complete and thorough assessment for common allergens was negative. During breastfeeding, for almost 6 months, Vit D supplementation was not provided. After the age of 5 years though, her dermatitis worsened, and the lesions spread all over her body (SCORAD 70) [15]. Intense scratching of the patient, which was very difficult to alleviate with the topical use of antibiotics, corticosteroids, and oral antihistamines, caused sleep disturbances and a huge frustration to the family (Figure 1(a)). All available treatments and protocols using local and systemic drug therapeutic options including calcineurin inhibitors and methotrexate had failed to control the disease. Narrowband ultraviolet B phototherapy, normally not recommended for <18 yrs of age, had not been offered, tried, or discussed by her treating dermatologists. Regarding family history of other atopic conditions, the older sister was diagnosed with mild atopic dermatitis. The mother claimed latex allergy, and the father mentioned he had unclear food allergy in his childhood. Nobody else in the family suffered from asthma or allergic rhinitis.

Upon review, we suggested a new measurement of TSH and several other laboratory tests including Vit D levels (Table 1).

We treated her with 0.5 mcg × 3/day calcitriol and 4000 IU/day cholecalciferol p.o. During treatment, no other medications except for local use of moisturizers were allowed. After two months, the patient's AD had improved significantly, and both the pruritus and the extent of the eczematous lesions were reduced. Then, we increased the dose to 1 mcg × 3/day calcitriol maintaining the cholecalciferol dose. At 6 months, there was almost healing of her skin with only mild existing dryness, no itch, and absent inflammation (SCORAD 10) (Figure 1(b)) with signs of postinflammatory pigmentation over her skin as well. Her quality of life had dramatically improved, and from complete depression such as dressed always veiled, experiencing constant fatigue, feelings of helplessness, pessimism, and hopelessness, sleeping too much and overeating, loss of interest in things once pleasurable, and persistent sad or “empty” feelings, she was joyful again resuming all sports and outdoor activities she ever dreamed of. We continued the same treatment controlling every 6 months the biochemical parameters related to calcium metabolism, and more specifically calcium (Ca), phosphorus (P), parathormone (PTH) in serum, and Ca and Ca/creatinine (Cr) in 24-hour urine samples. For one year, the values were within the normal range, and no adverse effects were detected. At 12 months, while on calcitriol 1 mcg x 3/day, we detected an increased Ca/Cr ratio in the urine for her age, and we decided to switch from calcitriol therapy to the “equivalent” as far as VDR activation is concerned with paricalcitol dose, that is, 2 mcg × 3/day. Written informed consent was obtained for the off-label use of paricalcitol. Clinically, the patient remained stable (Figure 1(c)) practically free of AD but hypercalciuria resolved completely (Table 1). Paricalcitol treatment is being continued for 2.5 years now with a 6-month follow-up. In the future, we will reassess a possible discontinuation of the treatment which for now is out of the question by the patient herself considering the stress, the psychological pressure, and the physical “disability” she experienced due to the disease in the past, expressing fear in any relevant discussion.

## 3. Discussion

We have reported the case of a girl with severe AD who had significant improvement—one could literally say almost healing—of her skin lesions within 6 months with calcitriol and subsequently with paricalcitol treatment, a result that remains with no relapses for more than 3 years now. While one cannot exclude the possibility of spontaneous remission, which is quite common in 8–12 years old atopic eczema patients, in our case, the clinical picture persisted and worsened for many years and clinical improvement to calcitriol therapy was imminent, minimizing this possibility.

Our patient had an extra risk factor for Vit D deficiency, considering that, as it has been shown in a study from Hata et al., high BMI and darker skin type in patients with AD are important risk factors for Vit D deficiency [16].

There are several studies that have linked vitamin D supplementation with either a decreased risk to develop AD or a clinical improvement of the symptoms of AD patients in the literature. Javanbahkt et al. assessed the potential treatment benefit of vitamin D supplementation in improving AD symptoms and found that administration of 1600 IU of oral vitamin D alone or in combination with vitamin E showed a significant improvement in SCORAD index as compared to placebo [17]. Sidbury et al. evaluated the effect of vitamin D in the form of 1000 IU of ergocalciferol daily supplementation on AD improvement and randomly assigned for vitamin D or placebo to eleven children with AD for 1 month. Although there was a beneficial effect in the treatment group, there was no statistically significant change in the mean of either group's AD clinical severity score [18]. Di Filippo et al. treated 39 children with AD with 1,000 IU/day oral vitamin D for 3 months and reported that vitamin D treatment may be an effective way for reducing AD severity, based on vitamin D serum levels and SCORAD index before and after treatment. More specifically, the Vit D supplementation normalized the pattern of Th1 and Th2 interleukin pattern [19]. However, in all the above studies, the vitamin D dose used was particularly low, inadequate even for prophylaxis from hypovitaminosis D, even more so to except a therapeutic effect and the duration too short to show results based on immunomodulation driven by a presumed rise in 1–25 OHD3 levels. Knowing that 25(OH) vitamin D suppresses macrophage adhesion and migration [20], we decided to use the active hormone calcitriol, whose immunomodulating properties have been shown even in negativating type 1 diabetes-associated autoantibodies [21], while properly substituting with 4000 IU cholecalciferol daily assured optimal (>40 ng/mL) 25 OHD levels [22], without any risk for overdosing [23].

As far as IgE levels are concerned, our patient had high IgE at the beginning and even higher during treatment with Vit D, indicating no obvious relevance between IgE levels and AD severity. However, according to Hyppönen et al., patients with low vitamin D (<10 ng/mL) or with high vitamin D serum levels (>54 ng/mL) have significantly higher IgE levels than healthy individuals (40–50 ng/mL), and consequent correction of serum concentrations of vitamin D reduces IgE level significantly [24].

Our decision to use paricalcitol, which has been proven safe for children older than 10 years, was based on its superiority compared to calcitriol, as far as the risk for increased serum calcium levels and urine calcium excretion is concerned while its immunomodulatory potency makes it a drug of interest in the therapy of chronic immune-mediated inflammatory diseases [25]. Of course, we took into serious consideration that the only known use of this drug is for secondary hyperparathyroidism in pediatric kidney disease, and we monitored our patient closely [26, 27].

In conclusion, calcitriol treatment may be considered for patients with severe or refractory AD, and its analog paricalcitol may prove an even safer treatment alternative as far as risk for hypercalcemia and especially hypercalciuria is concerned. In any case, Ca metabolism parameters should be closely monitored. Randomized controlled studies in children are required to prove the effectiveness and safety of this therapeutic approach, especially to establish the optimal dosage and type of Vit D administration.

## Figures and Tables

**Table 1:** Laboratory values at each visit.

	At presentation	6 months (calcitriol therapy)	1 yr (calcitriol therapy)	1.5 yr (paricalcitol therapy)	2 yr (paricalcitol therapy)	Normal range
BMI	28.32 (+2.94 SD)	27.80 (+2.77 SD)	27.78 (+2.70 SD)	28.21 (+2.71 SD)		kg/m^2^
TSH	4.9	1.71	11.35	4.8	3.16	0.60–4.84 *μ*IU/mL
FT4	1.14	1.31	1.22		1.12	0.9–1.9 ng/dL
PTH	37	18	12	23	31	10–65 pg/mL
P	4.3	4.5	5.6	5.5	5.6	3.5–5.5 mg/dL
Ca	9.6	10.1	10.0	10.2	9.9	8.5–10.5 mg/dL
1,25(OH)_2_D3	15.7	48	114	110	76	18–80 pg/mL
25(OH)D3	14	42.8	52.9	55.3	47.8	30–100 ng/mL
ALP	200	210	202	212	233	199–440 U/L
24 hr urine Ca		185	229	120	148	Female adults (or >50 kg body weight): <200 mg/24 hours
Urine Ca/Cr		0.21	0.27	0.22	0.17	<0.22%
IgE	120.5		271			<90 U/mL
